# Extracellular microvesicles from astrocytes contain functional glutamate transporters: regulation by protein kinase C and cell activation

**DOI:** 10.3389/fncel.2013.00251

**Published:** 2013-12-10

**Authors:** Romain-Daniel Gosselin, Patrick Meylan, Isabelle Decosterd

**Affiliations:** ^1^Pain Center, Department of Anesthesiology, University Hospital Center, and University of LausanneLausanne, Switzerland; ^2^Department Fundamental Neuroscience, University of LausanneLausanne, Switzerland

**Keywords:** glutamate transporters, extracellular microvesicles, astrocyte, glia, protein kinase C, nerve injury, spinal cord

## Abstract

Glutamate transport through astrocytic excitatory amino-acid transporters (EAAT)-1 and EAAT-2 is paramount for neural homeostasis. EAAT-1 has been reported in secreted extracellular microvesicles (eMV, such as exosomes) and because the protein kinase C (PKC) family controls the sub-cellular distribution of EAATs, we have explored whether PKCs drive EAATs into eMV. Using rat primary astrocytes, confocal immunofluorescence and ultracentrifugation on sucrose gradient we here report that PKC activation by phorbol myristate acetate (PMA) reorganizes EAAT-1 distribution and reduces functional [^3^H]-aspartate reuptake. Western-blots show that EAAT-1 is present in eMV from astrocyte conditioned medium, together with NaK ATPase and glutamine synthetase all being further increased after PMA treatment. However, nanoparticle tracking analysis reveals that PKC activation did not change particle concentration. Functional analysis indicates that eMV have the capacity to reuptake [^3^H]-aspartate. *In vivo*, we demonstrate that spinal astrocytic reaction induced by peripheral nerve lesion (spared nerve injury, SNI) is associated with a phosphorylation of PKC δ together with a shift of EAAT distribution ipsilaterally. *Ex vivo*, spinal explants from SNI rats release eMV with an increased content of NaK ATPase, EAAT-1 and EAAT-2. These data indicate PKC and cell activation as important regulators of EAAT-1 incorporation in eMV, and raise the possibility that microvesicular EAAT-1 may exert extracellular functions. Beyond a putative role in neuropathic pain, this phenomenon may be important for understanding neural homeostasis and a wide range of neurological diseases associated with astrocytic reaction as well as non-neurological diseases linked to eMV release.

## INTRODUCTION

Among a plethora of functions, astrocytes (the most abundant cells in the central nervous system) scavenge extracellular glutamate through membrane excitatory amino-acid transporters (EAAT-1 and EAAT-2). This reuptake is vital for neurotransmission (Danbolt, 2001). Decay or hyperfunction of the reuptake machinery have been identified or hypothesized in many neurological conditions as diverse as epilepsy, ataxia, amyotrophic lateral sclerosis, ischemia, schizophrenia, or neuropathic pain and their experimental models (Sung et al., 2003; Jen et al., 2005; Beart and O’Shea, 2007; Daemen et al., 2008; Chao et al., 2010; Gosselin et al., 2010b; Mirzaei et al., 2010). The current theory posits that the rate of transporter synthesis is not sufficient for EAAT regulation. The correct and stable anchoring of these transporters at the plasma membrane is a crucial modulation as well, in which the protein kinase C (PKC) family is deemed important (Susarla et al., 2004; Sheldon et al., 2008; Susarla and Robinson, 2008).

In addition to being present at the plasma membrane, EAATs have been shown recently to occur in extracellular microvesicles (eMV; Faure et al., 2006). These organelles of 40–1000 nm diameters may originate from intracellular compartments belonging to late endosomes and multivesicular bodies (these eMV are called exosomes, 40–100 nm) or from budding of plasma membrane (these eMV are referred to as shedding microvesicles, 100–1000 nm). Exosomes and shedding microvesicles have been found in virtually all investigated biological fluids and conditioned media. Microvesicles contain collections of proteins, RNA and lipids, which are highly conserved across cell types and listed in an online database (Mathivanan et al., 2012). The current theories posit that eMV are important cell-to-cell carriers for intercellular signaling molecules. Nevertheless, in spite of their possible biological importance, research on eMV is in its infancy with very little light shed on their regulation either in homeostasis or in pathology. In particular, the identification of intracellular signals that govern the sorting of a specific cargo protein to eMV is largely unresolved. Remarkably, although eMV have been classically considered important in the immune system and in tumor cells (Thery et al., 2009), they are now regarded as pleiotropic in cell-to-cell communication (Kharaziha et al., 2012) including in the nervous system where virtually all cell populations produce eMV (Fruhbeis et al., 2012).

The present study was therefore designed to address a twofold question. First, using primary cultures of rat astrocytes, we explored whether PKC activation regulates EAAT targeting to eMV. Second, using a model of spinal astrogliosis induced by peripheral nerve lesion we explored the possible deregulation of EAAT subcellular distribution and enrichment in eMV. Our results show that eMV from astrocytes contain functional EAAT-1 and that PKC activation results in a subcellular reorganization of EAAT-1 together with an enrichment of this same transporter in secreted eMV. Finally, *in vivo*, we confirm that spinal astrogliosis induced by peripheral nerve injury is associated with PKC activation, changes in EAAT-1 subcellular distribution (readdressing) as well as EAAT-1 and EAAT-2 enrichment into eMV.

## MATERIALS AND METHODS

### CULTURE OF PRIMARY ASTROCYTES AND MICROGLIA

Full cortical astrocytic cultures were used, according to the method of McCarthy with minor modifications (McCarthy and de Vellis, 1980, to maximize the yield of collected cells, thus minimizing the number of needed animals). Cerebral cortices from 2 day old Sprague Dawley rat pups were homogenized by mechanical trituration and seeded onto poly-D-lysine coated 75 cm^2^ flasks in Dulbecco’s modified eagle medium (DMEM) glutamax (Invitrogen, USA), 10% normal calf serum with penicillin/streptomycin (Invitrogen, USA). Cells were grown in a humidified 95% CO_2_ incubator at 37°C. At confluence, flasks were shaken at 250 rpm for 2 h. Detached microglia were seeded in coated 6-well microplates in culture medium supplemented with 30% astrocyte conditioned medium. Post-shaking adherent astrocytes were trypsinized and seeded at 50% confluence onto coated 6 or 24-well microplates or coverslips. Two days later, astrocytes were differentiated (culture medium, 2% normal calf serum, 150 μM dbcAMP, Sigma-Aldricht, USA) for 5 days. Pharmacological compounds were phorbol myristate acetate (PMA, final concentration 1 μM, Sigma, USA), Chelerythrine (CHE; 10 μM, 5 min pretreatment prior to PMA, Tocris, USA) or vehicle (1% DMSO in PBS, 5 min pretreatment prior to PMA).

### ANIMALS AND SURGERY

All procedures were approved by the Committee on Animal Experimentation for the canton of Vaud, Switzerland, in line with the Swiss Federal Law on Animal Welfare and the recommendations of the International Association for the Study of Pain. Spared nerve injury (SNI) was performed on male Sprague Dawley rats (Charler River, France) as previously described, on the left sciatic nerve (Decosterd and Woolf, 2000). In sham animals, the nerves were bared but left untouched.**

### SURFACE BIOTINYLATION

Confluent cells from 75 cm^2^ flasks were incubated for 30 min at 4°C with Biotin-solution (0.3 mg/ml in PBS, Thermo Scientific, Germany) and then washed twice in PBS. Cell were solubilized 1 h at 4°C, on agitation in lysis buffer (Hepes 50 mM pH 7.4, NaCl 150 mM, MgCl_2_ 1.5 mM, EDTA 1 mM, glycerol 10%, Triton X-100 1% and *N*-ethylmaleimide 1.2 mg/ml) supplemented with antiproteases. Homogenates were centrifuged (12,000 *g*, 15 min) and proteins were quantified in the supernatant using the Bradford method. 500 μg of proteins were incubated with 50 μl of streptavidin/Neutravidin-sepharose beads (GE Healthcare, USA) on agitation at 4°C and samples were centrifuged (1000 *g*, 2 min). S1 supernatants (corresponding to the non-biotinylated fractions) were supplemented with 5× sample buffer and stored at -20°C until use. Pellets were washed 5 times with lysis buffer supplemented with antiproteases and after the final wash, 2× sample buffer was added before incubation at 90°C for 5 min. Samples were centrifuged (3000 *g*, 5 min) and S2 supernatants (biotinylated fractions) were stored at -20°C until use. S1 and S2 fractions were processed for western-blot analysis.**

### SUBCELLULAR FRACTIONATION

Cells were mechanically resuspended in ice-cold 0.8 M sucrose/Tris 10 mM/pH 7.4 (300 μl, fractionation buffer) and submitted to 10 strokes through a 26 gauge needle. For *in vivo* experiments, performed at 7 days post-surgery, spinal dorsal halves were isolated, homogenized (motor pestle) in fractionation buffer (300 μl) and centrifuged at 1000 *g* for 10 min. Cell homogenates or spinal post-nuclear supernatants were loaded on top of a linear sucrose gradient (15–50%, in Tris 10 mM, pH 7.4, 3.2 ml in total) and centrifuged at 100,000 *g* for 2 h. Fourteen fractions (250 μl) were collected from bottom (high density) to top (low density) and stored at -80°C until use. For quantification of subcellular fractionation, the signal in each fraction was expressed as a ratio over total signal. All comparisons were made between samples that had been collected from the same series of cultures and ultracentrifuged together, to minimize the slight variations between different subcellular fractionations.

### ISOLATION OF MICROVESICLES

Microvesicles were isolated as previously described, with minor modifications (Caby et al., 2005; Alvarez-Erviti et al., 2011). Briefly, conditioned media from cells or spinal explants were centrifuged at 1500 *g* for 5 min and at 3000 *g* for 10 min to remove non-adherent cells, cell debris and apoptotic blebs. Supernatants were then ultracentrifuged at 100,000 *g* for 2 h and the microvesicular pellets were resuspended in western-blot loading buffer or phosphate buffer saline (PBS).

### NANOPARTICLE TRACKING ANALYSIS

Nanoparticle tracking analysis was performed using the Nanosight LM10-HS system (NanoSight, Amesbury, UK) as previously described with minor modifications (Soo et al., 2012). Briefly, eMV from astrocytes were prepared as described above and resuspended in PBS (1 ml of PBS for eMV corresponding to the conditioned medium from 75 cm^2^ of confluent astrocytes). Fifty microliters of the suspension were diluted 10 times in PBS for analysis (final volume 500 μl) and injected into the chamber of the apparatus. The technique employs a laser beam passing through the suspension of particles that are visualized by light scattering using a standard optical microscope. The Brownian motion of each particle is tracked between frames, and the Nanosight software calculates the size of each particle that passes in the field using the Stokes–Einstein equation.

### [^3^H] ASPARTATE REUPTAKE

Reuptake experiments on cultured astrocytes were conducted as follows. Cell medium was changed and astrocytes were first equilibrated for 5 min with incubation buffer (KCl 5.33 mM; NaHCO_3_ 26.2 mM; NaCl 117.2 mM; NaH_2_PO_4_ 1 mM; CaCl_2_ 1.2 mM; HEPES 1 mM; D-Glucose 5.6 mM; pH 7.4). Radiolabelled aspartate (50 nM final, 23.9 Ci/mmol, Amersham, Buckinghamshire, UK) was added and left to incubate for 5 min at RT. Cells were then washed three times with ice-cold incubation buffer to stop aspartate transport and lysed with 1 N NaOH + 0.05% sodium dodecyl sulfate in water. Radioactivity was estimated by liquid scintillation.

For [^3^H] aspartate reuptake on eMV, astrocyte medium was centrifuged for 5 min at 1500 *g* and 10 min at 3000 *g* before being supplemented with 20% incubation medium. Three samples were then incubated for 5 min with (2S,3S)-3-[3-[4-(trifluoromethyl)-benzoylamino]-benzyloxy]aspartate (TFB-TBOA, 100 nM final, generous gift from Dr. Jean-Yves Chatton; Bozzo and Chatton, 2010) and 3 samples were left untouched. [^3^H] aspartate (50 nM final) was added and left to incubate for 5 min at RT before media were ultracentrifuged 2 h at 100.000 *g*. The microvesicular pellet was then lysed in 1 N NaOH + 0.05% sodium dodecyl sulfate and the radioactivity counted by liquid scintillation.**

### WESTERN-BLOT

Western-blot was done as previously described (Gosselin et al., 2010a). The following antibodies were used (over-night, 4°C): rabbit anti-EAAT-1, EAAT-2, GAT-1 and GAT-3 (1/5000; Abcam, USA), caveolin-1 (1/200; Santa Cruz, USA), NaK ATPase β subunit (1/1000; generous gift from prof. Kaethi Geering), Iba-1 (1/1000; Wako, Japan), EEA1 (1/500; Cell Signaling, USA), mouse anti-glial fibrilary acidic protein (GFAP, 1/2000; Millipore, USA), NaK ATPase α subunit (1/1000; Abcam, USA), glutamine synthetase (1/5000; generous gift from Prof. Andrea Volterra), α-tubulin (1/10000; Sigma, USA) and Alix (1/2000; Abcam, USA). For phospho-PKC, rabbit monoclonal antibodies were used (1/1000; sampler kit, Cell Signaling, USA). For quantification, we used ImageJ freeware; quantification was made using Coomassie normalization for loading control.

### IMMUNOFLUORESCENCE

Immunofluorescence was performed as previously described (Gosselin et al., 2010a) on cryostat-cut 18 μm thick spinal sections from paraformaldehyde (PFA)-perfused rats or on PFA-fixed cells grown on coverslips. The following antibodies were used: rabbit anti-EAAT-1 (1/500; Abcam, USA), EAAT-2 (1/50; Abcam, USA), phospho-PKC (all antibodies 1/100), s100β (1/500; Sigma, USA), PGP9.5 (1/1000; Accurate Chemicals, USA), mouse anti-NeuN (1/300; Millipore, USA), glial fibrilary acidic protein (GFAP; 1/1000; Millipore, USA), glutamine synthetase (1/500; generous gift from Andrea Volterra). Nuclei were visualized by DAPI staining (5 min) in the first washing bath after secondary antibody. Digital images were all processed with the same settings to improve the contrasts with no particular part of any pictures modified independently. For evaluation of PMA toxicity, 6 fields per well from 3 wells of 24-well microplates (40× magnification) were used per condition using DAPI labeling.**

### SPINAL EXPLANTS

Under terminal pentobarbital anesthesia, rats were exsanguinated by decapitation and L4–L5 spinal cords were quickly isolated by laminectomy and collected in DMEM/glutamax (Invitrogen, USA), 10% normal calf serum with pennicillin/streptomycin (Invitrogen, USA). Tissues (approximately 5 mm in length) were then cut into 400 μm thick slices using a McIlwain tissue chopper (Mickle laboratories, UK). Explants were singly transferred in 500 μl of medium in 24-well microplates and incubated 24 h in a humidified 95% CO_2_ incubator at 37°C. Conditioned medium was pooled for each animal and processed for eMV isolation.**

### STATISTICAL ANALYSIS

All statistics were performed using GraphPadPrism software (version 5.01, GraphPad, USA); all data are expressed as mean ± S.E.M. For reuptake analysis, western-blot analysis of EAAT-1 in eMV and survival assay on cultured cells, one-way ANOVA was used, followed by Dunnett’s *post hoc* test vs. vehicle. For subcellular fractionation from cultured cells, two-ways ANOVA was used, followed by Bonferroni’s *post hoc* test. For reuptake on isolated eMV and for western-blot analysis of eMV from spinal explants, unpaired two-tailed Student’s *t*-test with Welch correction for unequal variances was used. For all other western-blot explorations, unpaired two-tailed Student’s *t*-test was used. For all statistics, threshold was set up at *p* < 0.05.**

## RESULTS

### PKC ACTIVATION MODIFIES SUBCELLULAR DISTRIBUTION OF EAAT-1

**FIGURE 1:**
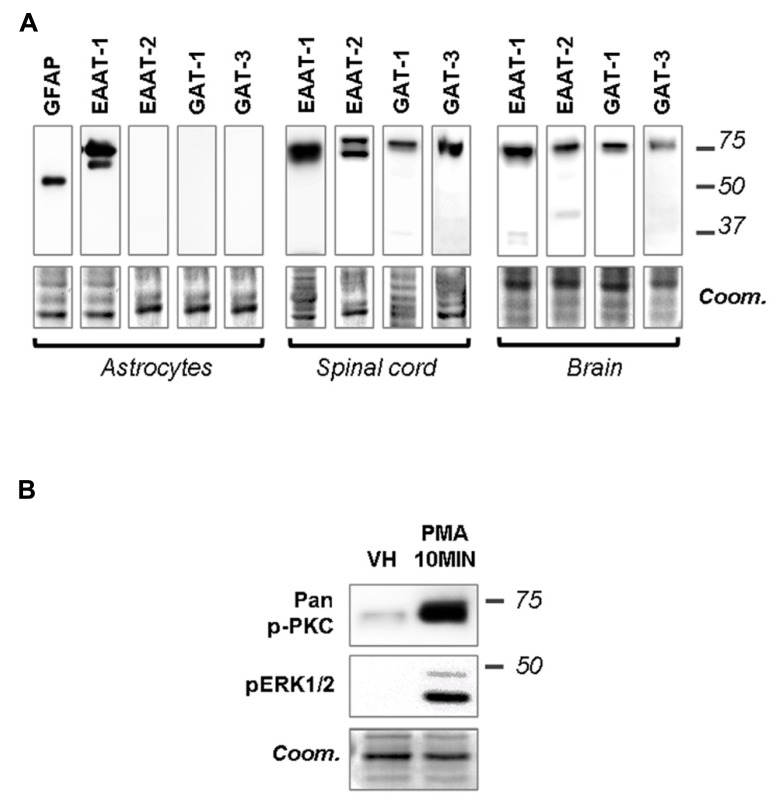
**Primary astrocytes express EAAT-1 and respond to PMA.** **(A)** Western-blots showing that EAAT-1 is detected in cultured astrocytes, as opposed to EAAT-2 or GABA transporters GAT-1 and GAT-3. Coomassie stain (coom) is shown in the lower panels as a loading control (similar images reflect multiple incubations of the same membranes with different antibodies). Signals obtained from dorsal spinal cord and brain are also shown as positive controls. Molecular weights (kDa) are given on the right. **(B)** Western-blot verifying the capacity of PMA (1 μM, 10 min) to activate the PKC pathway (pan-phospho PKC, upper panel) and downstream cascades (phospho ERK).

**FIGURE 2:**
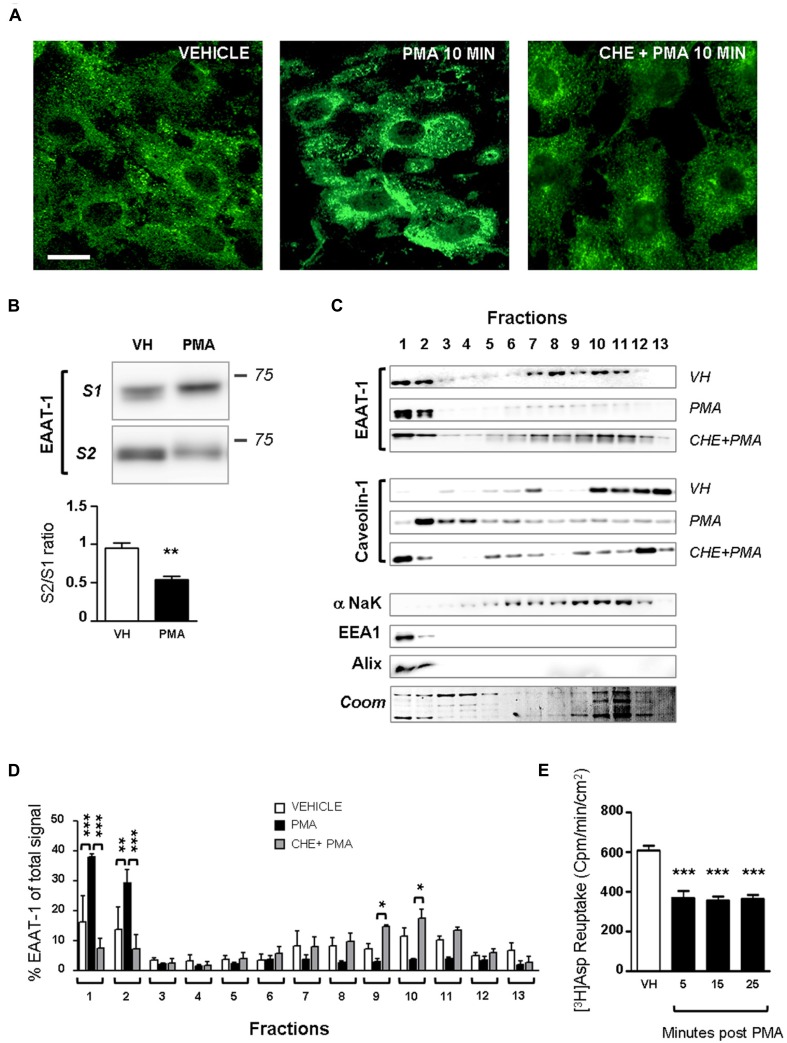
**PKC activation modifies EAAT-1 sub-cellular distribution in primary astrocytes.** **(A)** Fluorescence confocal microscopy showing the EAAT-1 immunoreactivity in vehicle-treated (left picture), PMA-treated (middle panel) and PKC inhibitor chelerythrine (CHE)/PMA-treated astrocytes (right picture). Scale bar: 10 μm. **(B)** Surface biotinylation of astrocytes showing the increased signal for internalized EAAT-1 (S1, non-biotinylated) and reduced surface EAAT-1 (S2, biotinylated) after PMA. One lane represents a homogenate from 75 cm^2^ confluent astrocytes. Quantifications were made from 5 blots corresponding to 5 independent flasks. **p < 0.01, Student’s *t*-test. **(C)** Subcellular fractionation by ultracentrifugation on sucrose gradient followed by western-blot showing that PMA treatment produces a relative enrichment of EAAT-1 in low density fractions 1–2 also rich in early (EEA1) and late (Alix) endosomes, as opposed to the reduction of EAAT-1 signal in plasma membrane (NaK ATPase) rich fractions 7–11. Pretreatment with CHE fully abolishes the EAAT-1 enrichment in fractions 1–2 both constitutively or upon PMA. **(D)** Bar histogram showing the quantification of EAAT-1 subcellular fractionation as a percentage of total signal. *p < 0.05; **p < 0.01; ***p < 0.001, two ways ANOVA, followed by Bonferroni *post hoc* test (*n* = 3 independent blots from 3 independent cultures). **(E)** [^3^H] Aspartate reuptake showing the inhibitory effect of PMA on the transport capacity of astrocytes (black bars) as compared to vehicle-treated cells (VH, white bar). ***p < 0.001 one way ANOVA followed by Dunnet’s *post hoc* test vs. vehicle column (*n* = 6 wells per condition).

Primary cultures of astrocytes from newborn rats were used to clarify the regulation of EAATs by PKCs. Quantification of cell markers in our culture by immunofluorescence gave the following proportions in astrocyte cultures: GFAP positive cells 45.8 ± 2.1% of total nuclei (72/160 nuclei in 5 fields, from a single culture); glutamine synthetase positive cells 74.5 ± 0.9% of total nuclei (135/183 nuclei in 5 fields); Iba1 positive cells 2.4 ± 0.8% of total nuclei (5/188 nuclei in 5 fields). In cultured astrocytes, western-blot analysis revealed EAAT-1 expression, as opposed to EAAT-2, GABA transporters (GAT)-1 and GAT-3, although all transporters were detected in spinal cord and brain samples (**Figure 1A**). We therefore focused our *in vitro* investigations on EAAT-1. Treatment with PMA elicited PKC activation as well as downstream ERK1/2 phosphorylation (**Figure 1B**). Confocal immunofluorescence revealed a profound reorganization of EAAT-1 immunoreactivity upon PMA exposure from a widespread signal throughout the cell to a highly condensed perinuclear cytosolic localization (**Figure 2A**). This PMA-triggered reorganization of EAAT-1 immunoreactivity was abolished by pretreatment with pan-PKC inhibitor CHE. Surface biotinylation shows that PMA treatment reduces the ratio between surface (S2) and intracellular (S1) EAAT-1 confirming the PKC-driven EAAT-1 internalization (**Figure 2B**). We further used ultracentrifugation on sucrose gradient (subcellular fractionation) to explore EAAT-1 subcellular distribution (**Figures 2C, D**). This technique allows the separation of cellular organelles according to their densities. The distribution pattern of EAAT-1 indicated a bimodal enrichment in fractions 1 to 2 and 7 to 11. Upon PMA treatment, a full leftward shift was observed for EAAT-1 signal, toward low-density with low protein level remaining in fractions 3 to 13. A shift toward low-density fractions was also observed for caveolin-1. Pretreatment with panPKC inhibitor CHE preserved EAAT-1 in fractions enriched in plasma membrane (significantly in fractions 9 and 10) and preserved low EAAT-1 in fractions 1–2. CHE pretreatment had only a mild effect on PMA-driven caveolin-1 readdressing, with a partial signal restoration in fractions 9–13 but without a full disappearance of caveolin-1 from fractions of lower density (**Figure 2C**). Analysis of organelle markers shows that fractions enriched in plasma membrane markers (sodium potassium pump, NaK ATPase: fractions 3 to 13; caveolin-1: fractions 5 to 7 and 10 to 13) were distinct from fractions containing markers for endomembranes (early endosomes, EEA-1: fraction 1; late endosomes, Alix: fractions 1 and 2). This distribution pattern suggests that in basal conditions EAAT-1 is distributed at both plasmalemmal and endomembrane sites and that upon PKC activation it is routed toward the endosomal compartment. Functionally, exposure to PMA was effective at significantly reducing [3H]-Aspartate reuptake from cultured astrocytes (**Figure 2E**). To explore the possibility that the observed PMA-driven EAAT-1 plasticity might be linked to a non-specific toxic effect, we examined whether cell death was provoked by PKC activation. No increase in the percentage of apoptotic nuclei could be found by morphological analysis of nuclear DAPI labeling 24 h (1.2 ± 1.0%) or 48 h (1.9 ± 0.8%) post-PMA as compared to vehicle-treated cells (1.6 ± 0.5%). Similarly, 24 h or 48 h PMA exposure did not reduce the total number of counted nuclei per field when compared to vehicle treatment (25.5 ± 1.5, 24.9 ± 1.4, 26.4 ± 1.3, respectively).

### CONSTITUTIVE AND PKC-SENSITIVE EAAT-1 ENRICHMENT IN ASTROCYTIC MICROVESICLES

**FIGURE 3:**
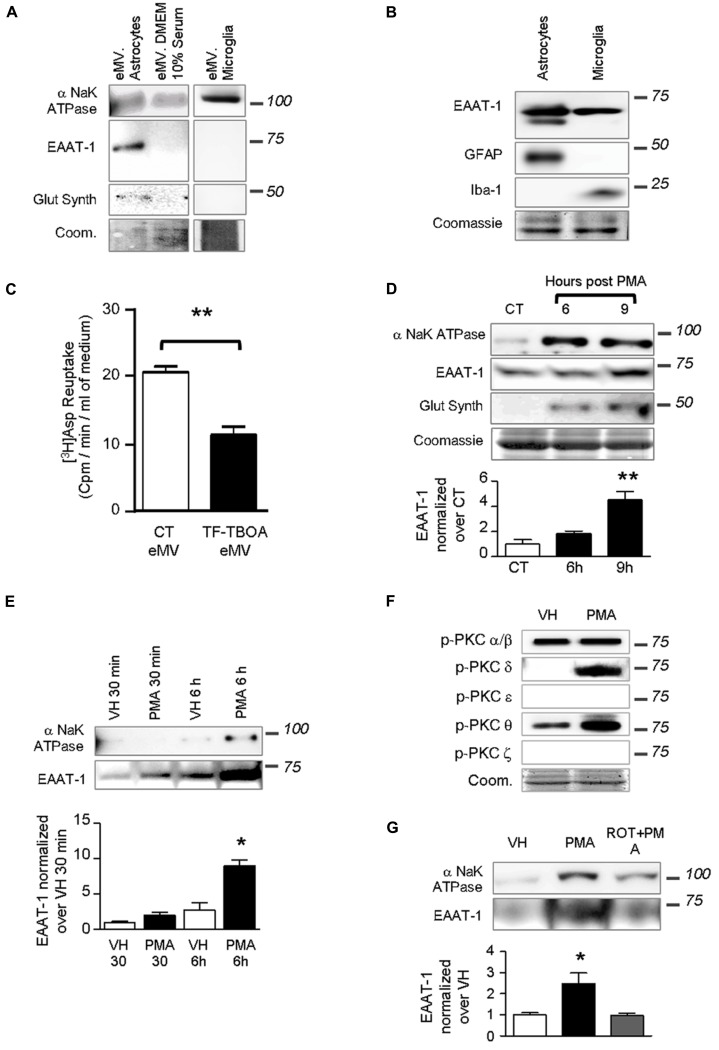
**Extracellular microvesicles from astrocytes contain functional EAAT-1.** **(A)** Representative western blots showing NaK ATPase, EAAT-1 and glutamine synthetase in astrocyte-derived eMV, whereas only NaK ATPase is detected in eMV from microglia or serum. Lanes correspond to medium from 75 cm^2^ of confluent cells. Coomassie staining (coom) shows total protein content. **(B)** Western blots of astrocyte (GFAP positive) and microglia (Iba-1 positive) extracts showing that both cell types express EAAT-1 in culture (10 μg of proteins extracts loaded). **(C)** Bar graph showing [^3^H] aspartate reuptake from astrocyte-conditioned medium treated (black bar) or not (white bar) with the specific EAAT blocker TF-TBOA. Given as mean ± SEM. **p < 0.001, *t*-test with Welch correction, *n* = 3 samples from 3 independent dishes. **(D)** Time course study of eMV composition in response to PMA without medium replacement. An enrichment of NaK ATPase and glutamine synthetase is found at 6 and 9 h post-treatment together with an increase of EAAT-1 signal after 9 h, but without modifications of Coomassie staining. Bar graph shows quantification of EAAT-1. **p < 0.01, one way ANOVA followed by Dunnett’s *post hoc* test vs. control column (*n* = 4 samples from independent cultures for each condition). **(E)** Time course study (30 min and 6 h) of microvesicular NaK ATPase and EAAT-1 in response to PMA after a full medium change. A significant augmentation of EAAT-1 is observed 6 h post-PMA in comparison to eMV from vehicle treated cells.*p < 0.05, one way ANOVA followed by Dunnett’s *post hoc* test vs. control column (*n* = 4 samples from independent cultures). **(F)** Western-blots showing the PKC isoforms activated in primary astrocytes after PMA treatment. PMA treatment increases phospho-PKC-δ and -θ. Coomassie staining (coom) shows the total protein content. **(G)** Western blot showing that rottlerin counteracts the increase of EAAT-1 in eMV after PMA. The bar histogram shows the quantification of band intensities. *p < 0.05, one way ANOVA followed by Dunnett’s *post hoc* test vs. control column (*n* = 3 independent cultures for each condition).

We investigated whether PKC activation could trigger the release of the transporter into eMVs. Conditioned cell culture media were ultracentrifuged and eMV-enriched pellets were analyzed (**Figure 3**). **Figure 3A** shows that both conditioned medium from astrocytes and microglia, as well as culture medium with serum, contained eMV as assessed by the detectable signal for membrane α subunit of NaK ATPase. xxx No signal for the β subunit of NaK ATPase could be found in eMV from cultured cells (not shown). EAAT-1 could only be detected in eMV isolated from astrocytic medium (**Figure 3A**), together with a weak but visible signal for glutamine synthetase. EAAT-1 expression could be detected in protein extracts from microglia as well, but not in microglia-derived eMV (**Figures 3A, B**). A reuptake experiment shows that a significant amount of [^3^H] aspartate can be incorporated in eMV and that this transport is sensitive to the selective EAAT blocker TFB-TBOA (**Figure 3C**), indicative of a functionality of eMV transporters.

Treatments of astrocytes with PMA for 9 h with no change of culture medium strongly increased the detectable signal for EAAT-1 (**Figure 3D**). Similarly, we found an increased signal for α-NaK ATPase and glutamine synthetase with no modification of Coomassie staining suggesting that protein targeting to the vesicles was modified with no change in the overall eMV release. *De novo* protein enrichment induced in eMV by PKC activation was assed by a full replacement of culture medium just prior treatment with PMA, followed by microvesicle isolations after 30 min or 6 h. This approach allows assessing the protein turnover in newly synthetized vesicles rather than a change in already released eMV. This procedure has induced an increase of EAAT-1 signal in eMV, which reached statistical significance only in samples treated 6 h prior to eMV collection (**Figure 3E**).

We investigated which of the different isoforms of PKC could be responsible for the regulation of EAAT-1 following PMA application. Marked phosporylations of PKC-δ and -θ were detected in response to PMA, with no effect on α/β or ε isoforms (**Figure 3F**). Pretreatment of astrocytes with rottlerin counteracted the significant increase of EAAT-1 in eMV induced by PMA (**Figure 3G**). The concentration of rottlerin that we employed is within the range of concentration allowing a selective inhibition of the PKC-δ isoenzyme with an IC50 of 3–6 μM against PKC-δ, as opposed to 30–100 μM for α, β, γ, ε, and ϖ isoforms (Gschwendt et al., 1994). PCK-ϖ was monitored to assess the specificity of activation, as the activation of this atypical isoform is insensitive to PMA.

**FIGURE 4:**
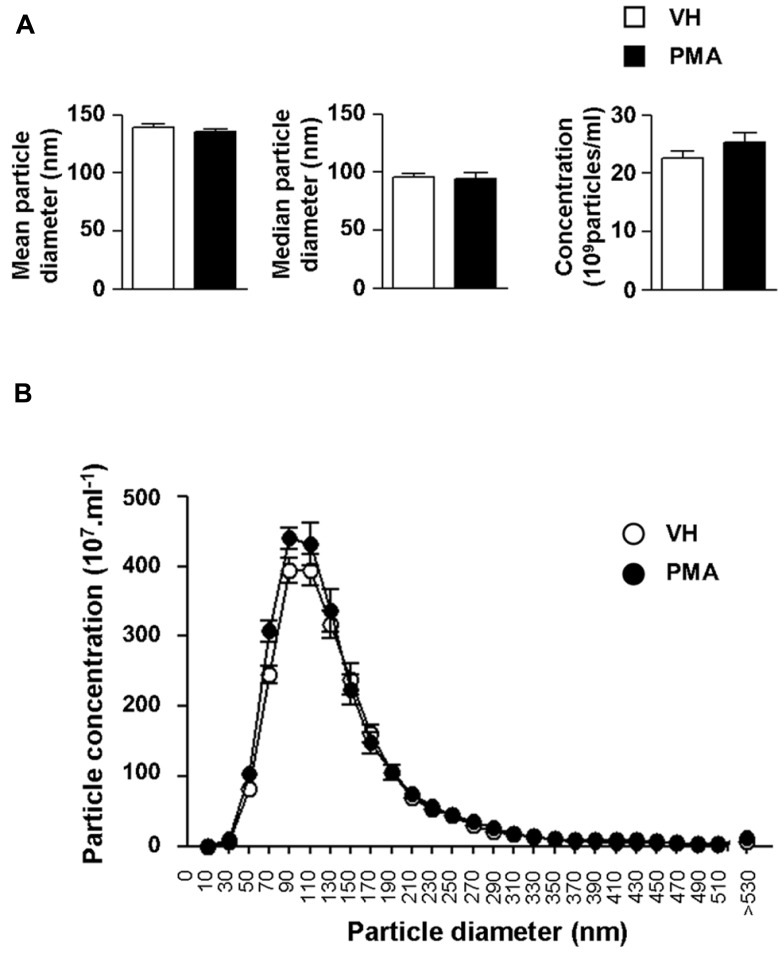
**Protein kinase C activation in primary astrocytes does not result in changes in eMV size or concentration.** **(A)** Bar graph showing the quantifications of mean diameter (left panel), median diameter (middle panel) and eMV concentration (right panel) in astrocytes conditioned medium 6 h after vehicle (white bars) or PMA (black bars) exposure. No significant difference could be found (Student’s *t*-test, *n* = 5 separate confluent flasks). **(B)** Size profile of eMV from vehicle (white circles) or PMA (black circles) treated astrocytes (*n* = 5 separate confluent flasks for each condition).

We further explored the structural properties of astrocytic eMV using laser-based nanoparticle tracking analysis (**Figure 4**). 6 h after PKC activation, we found no significant alteration of mean particle diameter (139.0 ± 3.1 vs. 135.4 ± 2.5 nm), median particle diameter (96.0 ± 2.7 vs. 94.0 ± 5.2 nm) or particle concentration (22.6 ± 1.2 vs. 25.2 ± 1.6 × 10^8^ particles ml^-^^1^) in conditionned medium (**Figure 4A**; *p* = 0.4048, 0.7408, 0.2237, respectively; Student’s *t*-test). In addition, we wanted to evaluate whether PKC activation generates subsidiary populations of vesicles that would not have significantly affected the global mean or median eMV diameter. Detailed analysis indicates that PMA exposure did not result in the emergence of secondary eMV populations with divergent diameter (**Figure 4B**). It is worth mentioning, however, that our isolation procedure might not be sensitive enough to distinguish biochemically divergent subpopulations of eMV with overlapping diameters.

### SPINAL ASTROGLIOSIS IS ASSOCIATED WITH PKC-δ ACTIVATION AND EAAT-1 REORGANIZATION

**FIGURE 5:**
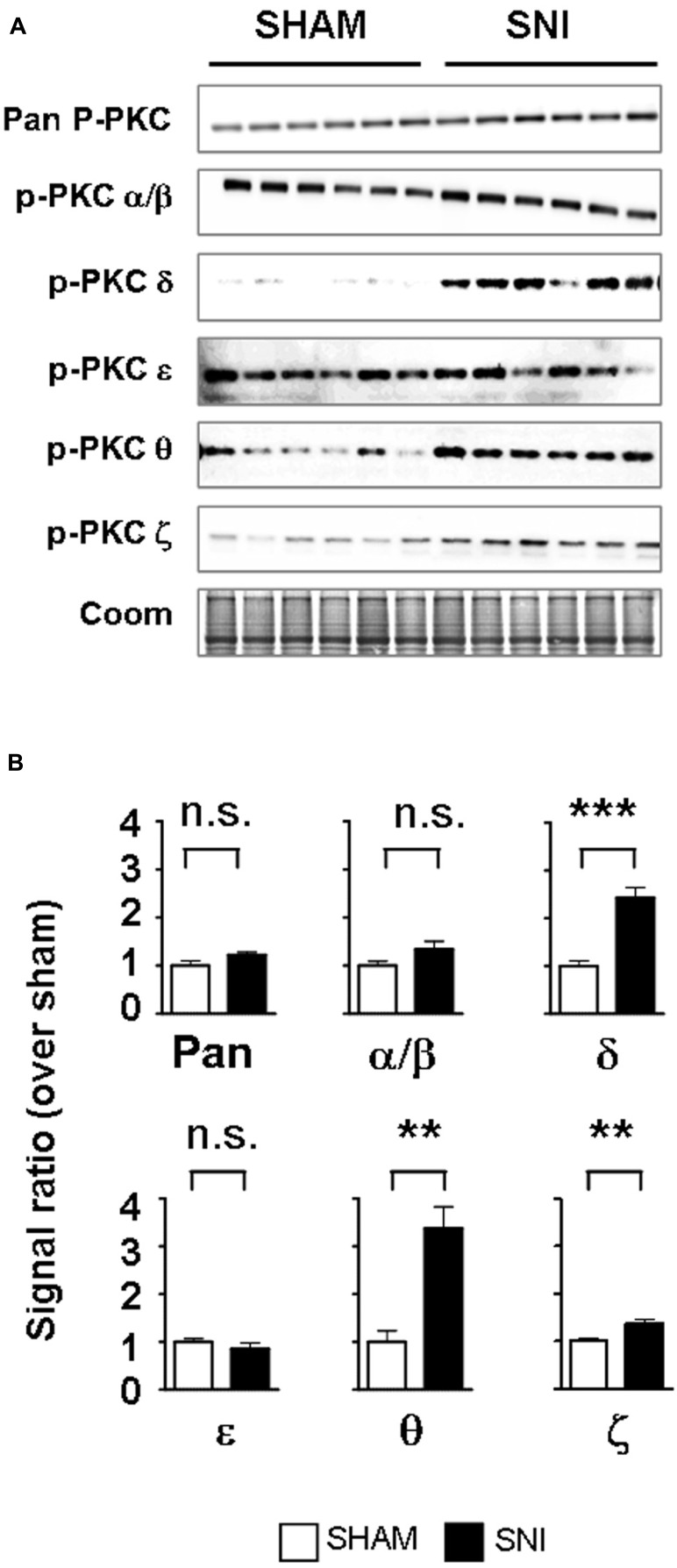
**Peripheral nerve injury induces the activation of PKC-δ, -θ and -ϖ in the spinal cord.** **(A)** Western blots showing phosphorylated PKC isoforms in the ipsilateral dorsal spinal cord of SNI and sham. **(B)** Bar graphs indicating the optical quantification of band intensity, as a ratio over Coomassie staining and normalized over sham, expressed as mean ± SEM. n.s., non significant; **p < 0.01; ***p < 0.001 *t*-test, sham vs. SNI, *n* = 6 for each condition. A significant activation of PKC-δ, -θ and -ϖ is found.

**FIGURE 6:**
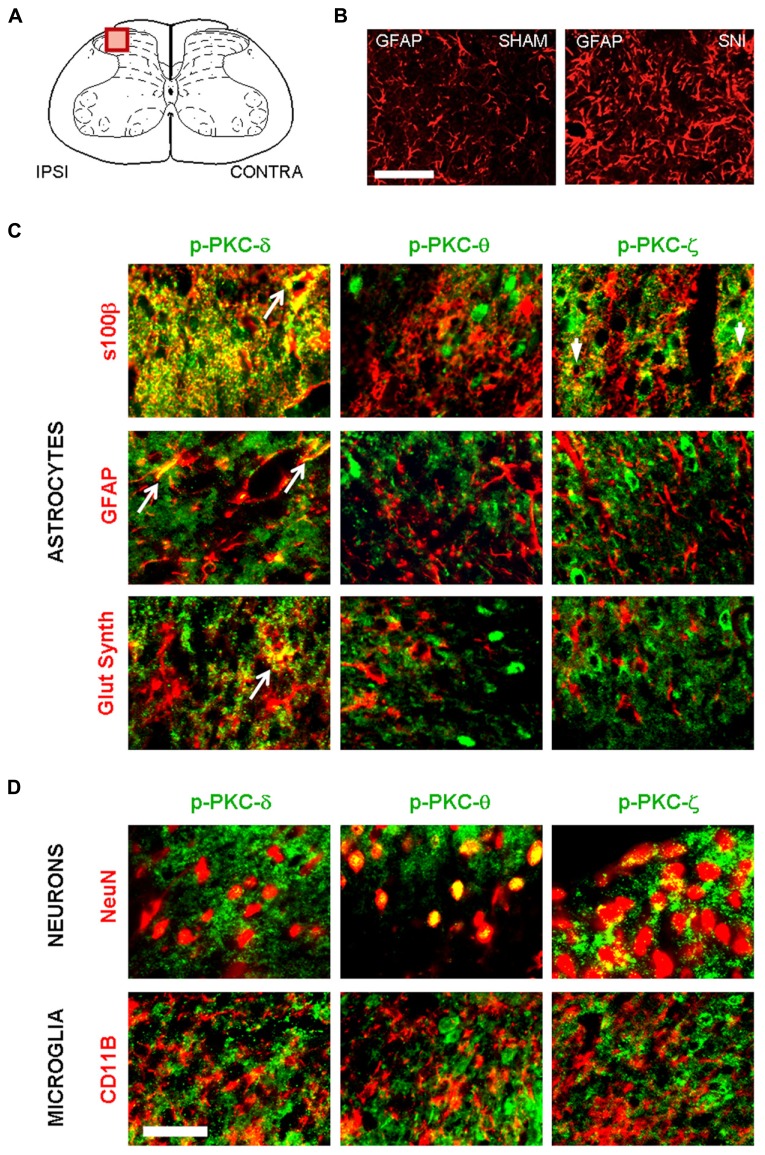
**Following peripheral nerve injury, astrocytes are the source of PKC-δ activation.** **(A)** Schematic showing the region of interest in the spinal dorsal horn. **(B)** Immunofluorescence highlighting spinal GFAP immunoreactivity one week after sham (left picture) or in SNI surgery (right picture). Scale bar 100 μm. **(B,C)** Immunofluorescence detecting isoforms of phospho PKC. **(C)** A colocalization is found between markers for astrocytes (s100β, GFAP and glutamine synthetase, red) and activated PKC-δ and -ϖ (green, first and third columns). No overlap was found between phospho PKC θ and astrocytes. **(D)** Phospho-PKC-θ and -ϖ, but not phospho-PKC-δ colocalizewith neuronal marker (NeuN, upper images). No isoform could be detected in microglia (CD11b, lower images). Scale bar, 100 μm.

We next determined whether PKC activation occurs *in vivo* during central astrogliosis induced by peripheral nerve injury. We used the SNI model of the sciatic nerve, which is well described for generating an astrocytic reaction in the ipsilateral spinal cord (Vega-Avelaira et al., 2007; Cirillo et al., 2011). After SNI, western-blots showed ipsilateral increases of phospho-PKC-δ (*p* = 0.0005), θ (*p* = 0.0023) and ζ (*p* = 0.0084) with no significant change in α/β or ε (**Figure 5**). Double labeling immunofluorescence (**Figure 6B**) showed that phospho-PKC-δ colocalizes with astrocytic markers S100β, GFAP and glutamine synthetase. Noteworthy, phospho-PKC-δ was found particularly in perivascular astrocytes. No colocalization was found between phospho-PKC-δ and neuronal markers NeuN or microglial protein CD11b (**Figure 6C**). Conversely, phospho-PKC θ colocalized with NeuN. Phospho-PKC-ζ was found mostly in subsets of neurons (**Figure 6C**) but also in scattered astrocytes (**Figure 6B**). None of these phospho-PKC isoforms was found to overlap with microglial marker CD11b (**Figure 6C**).

**FIGURE 7:**
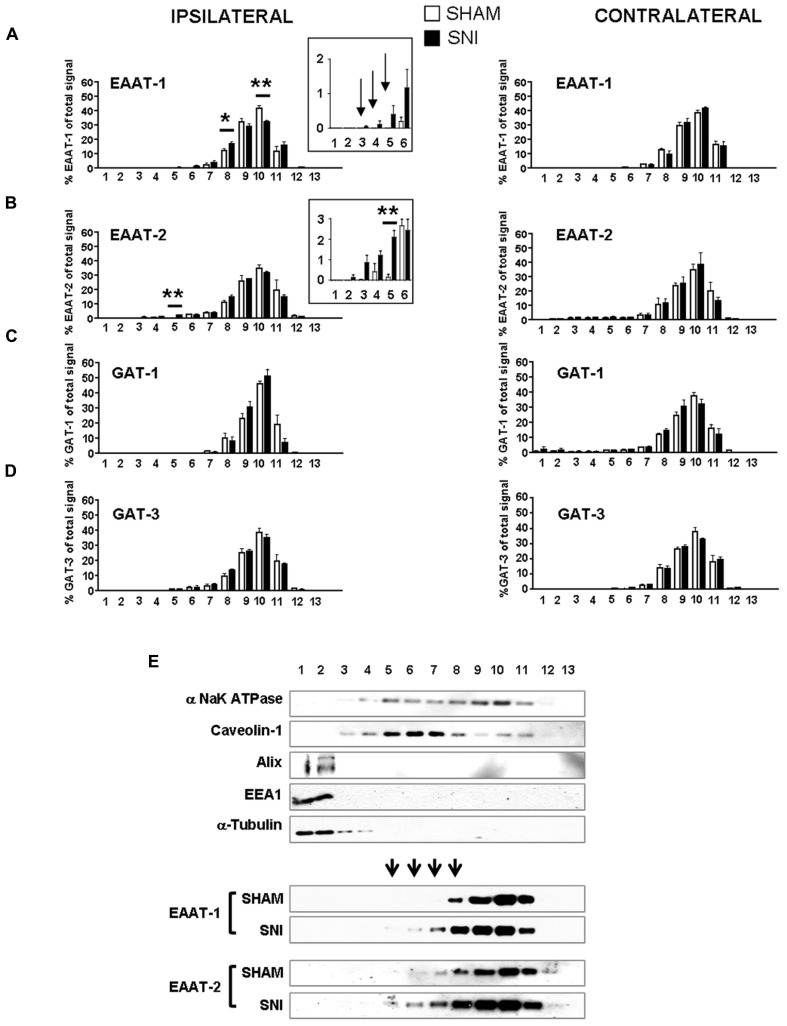
**(A–D)** subcellular fractionation presented as bar histograms, showing the distribution patterns of glutamate and GABA transporters as a percentage over total signal in all fractions. A leftward shift is observed for EAAT-1 **(A)** and EAAT-2 **(B)**. *p < 0.05; **p < 0.01, sham vs. SNI, *t*-test with Bonferroni correction for multiple comparisons (*n* = 5–6 rats for each condition). Arrows in **(A)** indicate signal detected in SNI samples with no counterparts in sham fractions. **(E)** Representative immunoblots of subcellular fractionation illustrating the shift of EAAT-1 and EAAT-2 content in SNI spinal cord, in the caveolin-1 enriched fractions. Arrowheads indicate fractions that are sites or EAAT-1 increase in SNI animals.

**FIGURE 8:**
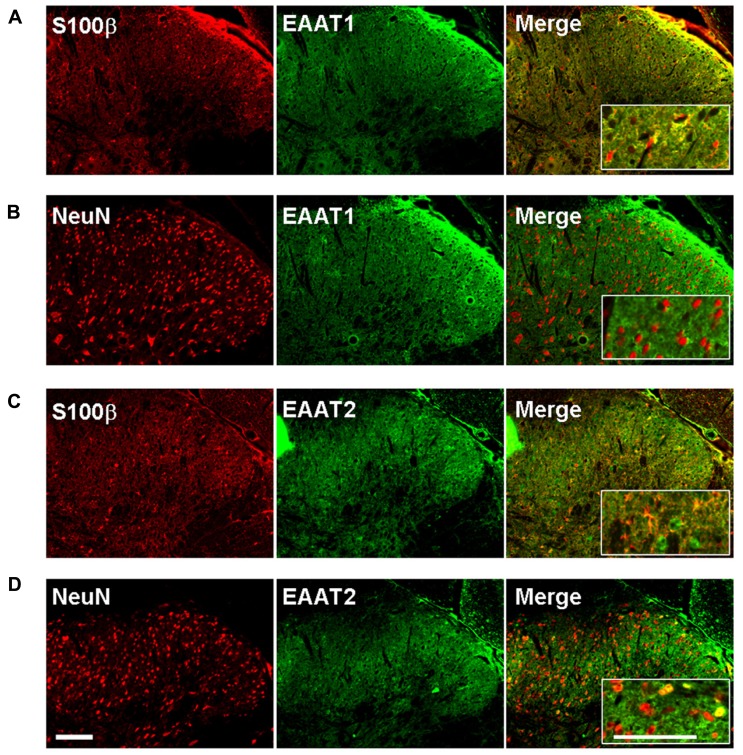
**EAAT-1 immunofluorescence is exclusively astrocytic.** **(A)** colocalization (yellow, right panel) between: EAAT-1 (green) and astrocytic s100β (red); **(B)** No colocalization between EAAT-1 (green) and neuronal NeuN (red). **(C,D)** EAAT-2 (green) is found in both astrocytes and some scattered neurons. Inserts show higher magnifications. Scale bar, 100 μm.

We therefore investigated whether EAAT-1 undergoes subcellular reorganization in the dorsal horn of the spinal cord after SNI (**Figure 7**). Astrogliosis resulted in modifications of EAAT-1 and EAAT-2 detected in subcellular fractionation, with a significant shift toward fractions of lower densities, containing caveolin-1. A significant increase in EAAT-1 signal was detected in fraction 8 (*p* = 0.0454), the protein became detectable in fractions 3 to 6, and a reduction of EAAT-1 content was found in fraction 10 (*p* = 0.0025; **Figures 7A, E**). A significant intensification of EAAT-2 occurred in fraction 5 (*p* = 0.0029) in SNI samples (**Figure 7B**). Remarkably, no EAAT modifications could be found in fractions 1 and 2, enriched in endosomal EEA1. There was no change on either side for NaK ATPase, GAT-1 or GAT-3 nor for EAAT contralaterally to SNI (**Figures 7A–E**), suggesting the specificity of ipsilateral EAAT subcellular plasticity following SNI. The amplitude of this shift is nevertheless much smaller than the modification obtained in primary astrocytes after PMA treatment (**Figure 1E**). We quantified EAAT-1 and EAAT-2 using western-blot of total extracts from ipsilateral spinal dorsal halves from sham and SNI rats (not shown). We found no statistical difference of EAAT-1 signal between the two groups of rats, whereas EAAT-2 was significantly increased in SNI animals (*p* = 0.5994 and 0.0127 for EAAT-1 and EAAT-2 respectively, *t*-test, *n* = 7 animals in each group). Finally, immunofluorescence showed a strong coincidence between EAAT-1 and the astrocytic protein s100β, but not with neuronal marker NeuN (**Figure 8**), supporting the astrocytic source of EAAT-1. Conversely, EAAT-2 immunoreactivity was found in subsets of both s100β and NeuN expressing cells, thus confirming the mixed source of EAAT-2 (de Vivo et al., 2010).

### SPINAL ASTROCYTIC ACTIVATION INCREASES THE eMV RELEASE OF EAAT-1 AND EAAT-2

**FIGURE 9:**
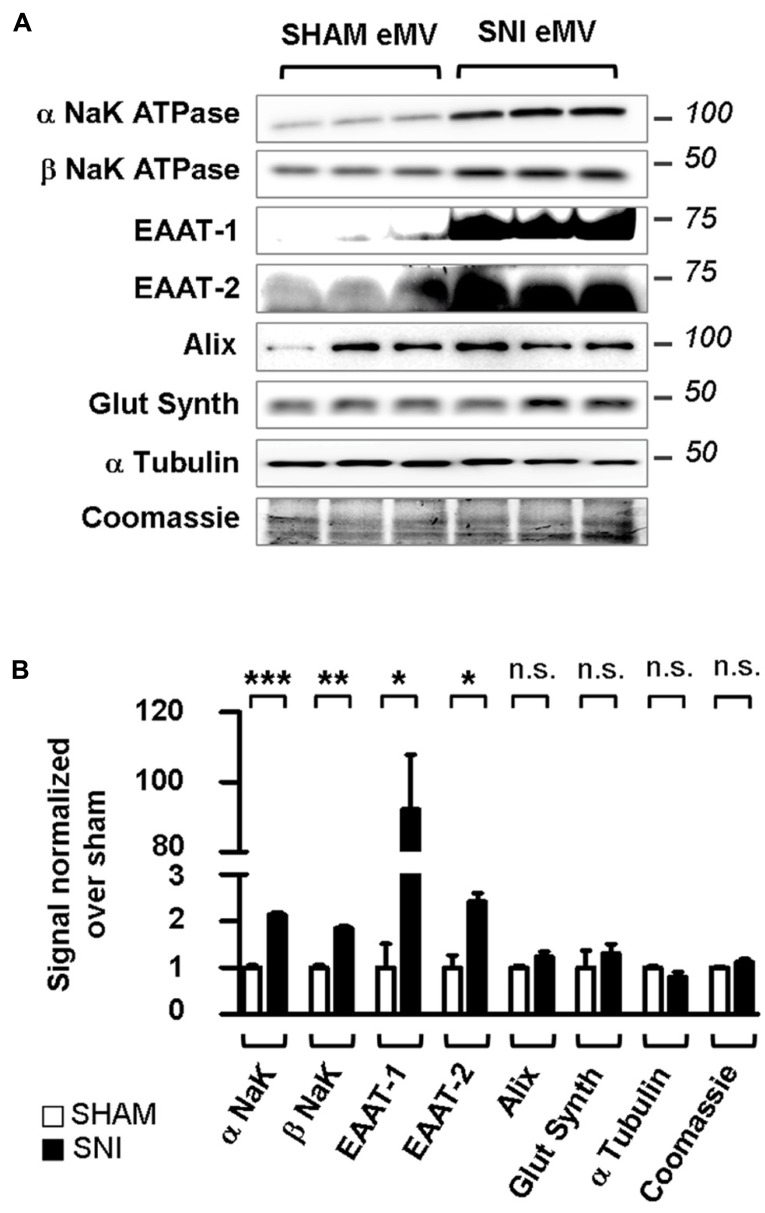
**Spared nerve injury spinal explants are enriched in EAAT-1 and EAAT-2.** **(A)** Western blots illustrating the increased NaK ATPase, EAAT-1, EAAT-2 but not glutamine synthetase, Alix, α tubulin or total protein content (Coomassie) contents in eMV from SNI rats as compared to counterparts from sham animals. **(B)** Quantification of western blot signals confirming the statistical increases observed in **(A)**. Given as mean ± SEM. *p < 0.05; **p < 0.01; ***p < 0.001; n.s., non significant. Student’s *t*-test with Welch correction for unequal variances, sham vs. SNI (*n* = 3 explants from different animals per condition).

The PKC activation and EAAT-1 subcellular reorganization generated during spinal astrogliosis suggest that an enrichment of EAAT in eMV might occur in parallel. We therefore characterized the eMV released by spinal explants from sham or SNI rats. In conditioned medium from spinal explants from sham animals, both α and β subunits of NaK ATPase were detected as well as glutamine synthetase, Alix, α tubulin and EAAT-2 as opposed to very low levels of EAAT-1 (**Figure 9A**). In conditioned media released by explants from neuropathic animals, a significant increase of α/β NaK ATPase (*p* = 0.0005 and 0.0012, respectively), EAAT-1 (*p* = 0.0285) and EAAT-2 (*p* = 0.0217) were found (**Figure 9B**). No change of glutamine synthetase, Alix or α tubulin contents could be detected (*p* = 0.1715,). In addition, we found no apparent modification of total protein content between samples from sham or SNI explants, quantified by Coomassie staining. Nonetheless, it has been shown (Verderio et al., 2012) that eMV derived from all neural cells can be detected for instance in the cerebrospinal fluid. We therefore cannot fully exclude that the observed changes stem from eMV released by non-astrocytic cells.

## DISCUSSION

**FIGURE 10:**
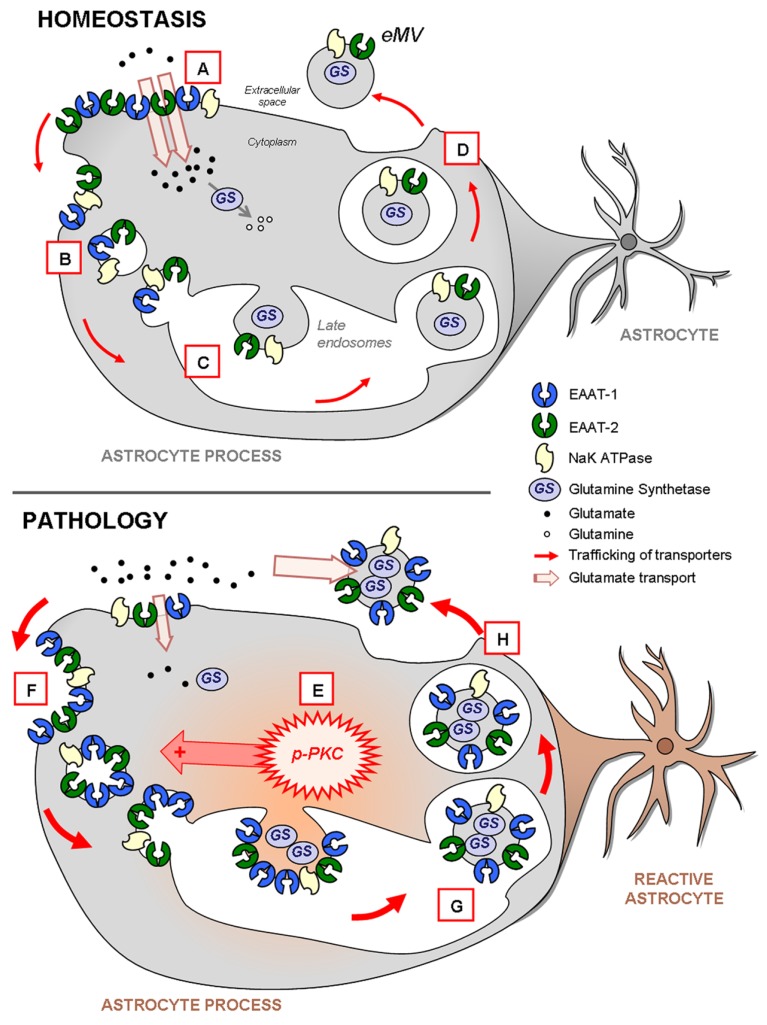
**Schema highlighting the trafficking of glutamate transporters in resting or reactive astrocytes.** In homeostatic conditions (upper panel), both EAAT-1 and -2 are enriched at the plasma membrane providing an efficient glutamate reuptake (**A**; see **Figures 2 and 7**) as well as in intracellular early **(B)** or late **(C)** endosomal pools (**Figure 2**). Furthermore, EAAT are also constitutively targeted to eMV (**D**; see **Figure 3**) together with glutamine synthetase and NaK ATPase, especially EAAT-2 *in vivo* (see **Figure 9**). During astrocytic reaction associated with pathological conditions (lower panel; see **Figure 6**) the activation of PKC (**E**; see **Figure 2**) and in particular PKC-δ (see **Figures 3, 5, and 6**) drives the internalization of EAAT to the endosomal compartment (**F**; see **Figures 2 and 7**). The result is an apparent loss of astrocytic reuptake capacity (see **Figure 2**). This PKC-driven EAAT internalization ultimately leads to increased transporter packing **(G)** and secretion in eMV **(H)** together with glutamine synthetase and NaK ATPase (**Figures 3 and 9**). However, no overall increase of eMV release is generated (**Figures 3, 4, and 9**). The ability of eMV to reuptake glutamate (see **Figure 3**) together with the concomitant presence of glutamine synthetase and NaK ATPase (see **Figure 9**) might bestow an increased extracellular capacity to convert glutamate to glutamine.

Our major result is that in astrocytes the enrichment of EAAT-1 in eMV is under the control of PKC. The activation of PKCs results in a reduction of the astrocytic glutamate transport, associated with a profound modification of EAAT-1 subcellular distribution toward both an intracellular pool and secreted microvesicles. Additionally, we suggest that the increased EAAT in eMV following PKC activation is a consequence of a selective routing of transporters to the microvesicular compartment rather than an increased number of secreted eMV. Moreover, glutamate transporters in astrocytic eMV are functional and we show that astrocytic reaction results in both a subcellular reorganization of EAAT and an increased EAAT content in released eMV (**Figure 10**).

The biochemical signals driving the enrichment of a cargo protein to eMV are a subject of current investigation but the available data remain scant (Bobrie et al., 2011; Hurley and Odorizzi, 2012). Nevertheless, the control exerted by PKCs on the circulation of EAAT-1 between the plasma membrane and intracellular pools has been demonstrated (Susarla et al., 2004; Sheldon et al., 2008; Susarla and Robinson, 2008). Our present study is, to our knowledge, the first to demonstrate that PKCs also govern the routing of EAAT-1 to eMV. Little is known about the signaling pathways regulating the release of eMV. One important identified pathway is the cascade involving sphingomyelinases and leading to the production of ceramides (Trajkovic et al., 2008; Yuyama et al., 2012), especially in astrocytes (Wang et al., 2012). Interestingly the PKC family, and particularly PKC-δ, has been identified as an important activator of sphingomyelinases and ceramide generation (Zeidan and Hannun, 2007; Clarke et al., 2008), which prompts further complementary exploration of a possible involvement of this pathway in EAAT sorting in astrocytic eMV. Besides, our results suggest that PKC drives subcellular reorganization of EAAT-1 and caveolin-1 in comparable fractions. This putatively points to a co-segregation of EAAT-1 into caveolae, a caveolin-rich form of lipid rafts (Lajoie and Nabi, 2010), in which EAAT have been previously detected (Escartin et al., 2006). The functional consequences of the connection between EAAT-1 and caveolin-1 are unclear. Nevertheless, numerous convergent studies establish both that eMV (exosomes and shedding microvesicles) contain rafts components and that eMV may arise from caveolae or lipid rafts (de Gassart et al., 2003; Del Conde et al., 2005; Chen et al., 2011; Valapala and Vishwanatha, 2011). It is however worth mentioning that CHE has dissimilar effects on PMA-induced re-addressing of EAAT-1 and caveolin-1, suggesting that the control exerted by PKC on EAAT-1 sorting extends beyond the sole incorporation in caveolae. It is worth pointing that our objective was to detect and quantify eMV release from astrocytes, and that the full description of eMV heterogeneity is not assessed in our experiments. Future studies using more refined isolation protocols may add new information about different populations of eMV, especially whether the released vesicles comprise exosomes or shedding microvesicles. This investigation was however, beyond the scope of our present study.

Our cultured neonatal astrocytes express EAAT-1 and not EAAT-2, thus all our present *in vitro* explorations were oriented toward the description of EAAT-1 regulation upon PKC activation. Nevertheless, the detection of EAAT-2 in eMV secreted by spinal explants after nerve injury suggests that mature astrocytes in their native cytoarchitectural environment address EAAT-2 to eMV as well. Remarkably, the previously described regulation of EAAT-2 subcellular sorting by PKC pleads in favor of a similar PKC-driven control of EAAT-2 addressing to eMV (Kalandadze et al., 2002; Sheldon et al., 2008; Susarla and Robinson, 2008). We found that the spinal astrocytic reaction generated after peripheral nerve injury was associated with: (i) the phosphorylation of PKC-δ and θ; (ii) a shift of glutamate transporters EAAT-1 and EAAT-2 from fractions with high contents of NaK-ATPase toward intermediate densities, with enrichment in caveolin-1; (iii) and an increased EAAT-content in eMV. No change was observed for GABA transporters GAT-1 and GAT-3 suggesting that EAAT reorganization is selective. Remarkably, the magnitude of EAAT-1 subcellular reorganization *in vivo* is lower than in PMA-treated astrocytes, suggesting that a limited number of astrocytes undergo such plasticity in SNI. Additionally, this phenomenon might be limited *in vivo* to a restricted percentage of EAAT-1 proteins within the cells.

Excitatory amino-acid transporters uses the electrochemical plasmalemmal gradient of Na^+^, generated by the NaK ATPase, to transport glutamate that will subsequently be converted into glutamine by glutamine synthetase. EAAT and NaK ATPase even physically assembly into a multi-protein complex, thus promoting functional coupling between glutamate transport and plasmalemmal ion flux (Rose et al., 2009). Therefore, the detection of this full protein arsenal in eMV from astrocytes suggests that microvesicular EAAT might have a functional extracellular role in glutamate elimination. Notably, we did not detect the regulatory β subunit of NaK ATPase in astrocyte-derived eMV, suggesting that another subunit might act as a surrogate interacting partner for α NaK ATPase as previously described (Horisberger et al., 1991). In accordance with the theory of an eMV-based glutamate detoxifiation, we found that eMV have the capacity to reuptake [^3^H] aspartate, indicating that microvesicular EAAT are functional. This could constitute an important conceptual breakthrough, as eMVs are widely considered important in intercellular communication, but practically unexplored for possible extracellular roles (Mathivanan et al., 2010). In support of this hypothesis, the careful exploration of the Exocarta compendium reveals that beyond EAAT, a wide range of membrane transporters has been detected in eMV from multiple origins. These include transporters for glucose, nucleosides, urate, peptides or amino acids mostly belonging to the solute carrier family (SLC), which also encompass EAAT. Strikingly, other transporters for neurotransmitters are found in this list, such as serotonin and γ amino-butyric acid (GABA) transporters, suggesting that extracellular reuptake functions of eMV might extend beyond glutamate.

The increase of EAAT and NaK ATPase in eMV during neuroinflammatory astrogliosis might therefore have functional consequences through an enhancement of the buffering capacity for extracellular glutamate. This could constitute a compensatory mechanism in response to increased glutamate release and more broadly as a consequence of a rise in glutamatergic transmission, as encountered following peripheral nerve injury (Larsson, 2009; Larsson and Broman, 2011). Further in-depth investigations are required to establish whether NaK ATPase, EAAT and glutamine synthetase coexist in the same microvesicles or are simultaneously released in different pools of eMV. In particular, as we also found EAAT-2 immunoreactivity in neuronal cells, it is possible that this transporter is partly released through a neuronal pool of eMV. Nevertheless, one cannot rule out the possibility that EAAT-containing eMV may play a role in intercellular communication in addition to extracellular glutamate buffering. The question therefore arises of the biological significance of a trans-cellular transfer of glutamate transporters. One may hypothesize that astrocytes synthetizing high amounts of EAAT initiate an eMV-mediated transfer of these proteins to cells expressing low rates of transporters, thus allowing a harmonization of parenchymal reuptake. Alternatively, reactive astrocytes might reduce their ability to scavenge synaptic glutamate through the reorientation of EAAT from the plasma membrane surrounding the synaptic cleft toward secreted eMV and a further extracellular pool destined to trans-cellular degradation. The short time-scale (minutes) of such regulation might constitute an advantage over transcriptional regulations that may necessitate hours to functionally echo on glutamate transport.

Peripheral nerve lesions, in particular the SNI model used in this work, are often used as models of chronic neuropathic pain (Decosterd and Woolf, 2000). These models are associated with a well-described astrocytic reaction in the spinal cord that is important in the perpetuation of sensory deregulations, in particular those due to alterations of EAAT (Gosselin et al., 2010b). However, astrocytic reactions take place in a wide variety of neurological conditions indicating that the deregulation of astrocytic eMV might have far-reaching consequences in neuropathology. Neuroinflammatory diseases such as multiple sclerosis represent typical conditions for which a better understanding of eMV release from glia might provide critical insights for innovative treatments. The well-described glial reactions, including also microglial and oligodendroglial origin, that take place in multiple sclerosis (Lassmann, 2011; Sriram, 2011) as well as the associated alteration of EAAT expression (Vercellino et al., 2007) both support this prediction. In line with this hypothesis, several studies have pointed at glial eMV in multiple sclerosis (Scolding et al., 1989; Verderio et al., 2012). Furthermore, glia-derived eMV might be future targets in other neurological conditions associated with profound gliosis and EAAT dysfunction such as stroke or spinal cord injury (Han et al., 2008; Ketheeswaranathan et al., 2011). Besides the established neuro-inflammatory diseases listed above, eMV constitute a promising field of investigation in neurodegenerative diseases as well, so far mostly explored under the angle of neuronal eMV (Rajendran et al., 2006; Gomes et al., 2007; Emmanouilidou et al., 2010; Saman et al., 2012). Besides, even though we do not provide direct evidence that such PKC-driven regulation also occurs in homeostasis, the presence of EAAT in eMV from non-stimulated cells and explants suggests this possibility. However, despite the above-mentioned medical potential, significant breakthroughs will be obstructed by the persisting lack of consensual classifications and definitions regarding eMVs. In depth investigations about eMV regulations are urgently needed to both shed some significant light on their function in homeostasis, as well as to convert these insights into credible clinical outcomes.

Altogether, our data on EAAT release in eMV add a new dimension to the regulation of glutamate transporters in astrogliosis. This may open new avenues for neurological treatments involving eMV regulation by PKC. Alternatively, exogenous interventional strategies using the capacity of eMV to scavenge glutamate may ultimately provide a way to address pathologies connected with defects in EAAT function.

## Conflict of Interest Statement

The authors declare that the research was conducted in the absence of any commercial or financial relationships that could be construed as a potential conflict of interest.

## AUTHOR CONTRIBUTIONS

Romain-Daniel Gosselin designed the experiments, performed the majority of the experiments and analysis, wrote the manuscript. Patrick Meylan carried out Immunoblot Blot assays and analysis for glutamate transporters. Isabelle Decosterd provided input in the design of the experiments, supervised the experiments and corrected the manuscript.
